# The Gut–Liver Immune Axis in Celiac Disease, Primary Sclerosing Cholangitis, and Ulcerative Colitis: Outcomes of a Rare but Significant Triad

**DOI:** 10.21203/rs.3.rs-11064634/v1

**Published:** 2026-10-04

**Authors:** Samruddhi Nitin Patwardhan, Omid Yazdani, Romina Esbati, Srusti Ganesh Bhalerao, Shaikh Danish Mahmood, Ana Marenco, Natalia Rojas-amaris, Joshua R. Korzenik

**Affiliations:** Brigham and Women's Hospital; Brigham and Women's Hospital; Brigham and Women's Hospital; Brigham and Women's Hospital; Brigham and Women's Hospital; Beth Israel Deaconess Medical Center; Beth Israel Deaconess Medical Center; Brigham and Women's Hospital

**Keywords:** Gluten-Free Diet, Liver Transplantation, Cirrhosis, Autoimmune Hepatitis

## Abstract

**Purpose:**

To characterize the clinical presentation, disease course, and outcomes of patients with concurrent primary sclerosing cholangitis (PSC) and celiac disease (CeD), including those with concomitant inflammatory bowel disease (IBD).

**Methods:**

Retrospective EHR analysis (MGB RPDR, BIDMC Celiac Center, All of Us) identified patients with confirmed PSC and CeD (ICD-10, encounter threshold > 1, validated clinically/serologically/histologically). Primary outcomes were hepatic complications and time to liver transplantation, compared against two 3:1 age-matched cohorts (PSC + IBD, n = 54; PSC-only, n = 54).

**Results:**

Among 7.5 million patients in RPDR, 18,375 had CeD and 992 had PSC, with 13 observed cases of overlap versus 2.43 expected (OR 5.35, 95% CI 3.08–9.30; p < 0.001). Eighteen patients met full inclusion criteria (67% male, 94% White, mean age 47.8 years at PSC diagnosis). IBD was present in 72%, predominantly Ulcerative Colitis (56%). With high gluten-free diet adherence (89%), hepatic complications occurred in 89% of patients, with cirrhosis in 50%. Liver transplantation was required in 22%, at a mean of 5.4 years from PSC diagnosis among transplants with dated intervals, with no post-transplant PSC recurrence observed among the 4 transplanted patients. Compared with matched PSC + IBD (n = 54) and PSC-only (n = 54) control cohorts, liver transplantation was numerically more frequent in the PSC + CeD cohort than in either control cohort, though this difference did not reach significance (22.2% vs 5.6% vs 13.0%; chi-square p = 0.127).

**Conclusion:**

PSC and CeD co-occur at a significantly higher frequency than expected, suggesting shared autoimmune pathogenic mechanisms. The substantial hepatic complication burden and frequent IBD co-occurrence highlight the clinical importance of this overlap and the need for multidisciplinary management and routine autoimmune screening in affected patients. Matched-cohort comparisons further suggest a numerically elevated, though not statistically significant, risk of liver transplantation in PSC + CeD patients relative to PSC + IBD patients without celiac disease, alongside a significantly elevated risk of cirrhosis and PSC–autoimmune hepatitis overlap.

## Introduction

The coexistence of multiple immune-mediated diseases within a single patient is increasingly recognized as a clinically significant phenomenon, yet the implications of such overlap often remain poorly understood both with regard to insights into clinical care and potentially disease pathophysiology. Among these, the intersection of celiac disease (CeD) and primary sclerosing cholangitis (PSC) presents a compelling but underexplored area of study. In addition, as PSC most often accompanies ulcerative colitis [1], consequently for many individuals, this represents an overlapping occurrence of three autoimmune diseases. While each condition is individually associated with autoimmune dysregulation and gastrointestinal morbidity, their concurrent presence raises important questions about shared pathophysiological mechanisms and compounded clinical burden. Case reports and small series have hinted at a potential association, suggesting that undiagnosed celiac disease may exacerbate malabsorptive symptoms or alter the disease trajectory in PSC [2]. However, the absence of larger, outcome-driven studies leaves clinicians without clear guidance for managing this overlap.

Both PSC and CeD are chronic immune-mediated disorders with overlapping pathogenetic features. PSC is an autoimmune cholestatic liver disease often linked with Inflammatory Bowel Disease usually UC [1], and celiac disease is an HLA (Human Leukocyte Antigen) -associated enteropathy triggered by gluten [3]. Familial reports hint at a shared predisposition: for example, two sisters with ulcerative colitis, PSC, and silent celiac disease both carried HLA-DR3/DQ2 risk haplotypes [4]. Early case series also noted a suspected immunologic connection documented three PSC patients who concurrently had celiac disease and malabsorption [9]. In those cases, initiating a gluten-free diet reversed steatorrhea but did not alter the underlying cholangiopathy [9]. Such observations suggest that these diseases may share immune pathways (e.g. gut-derived immune activation) even though their precise relationship remains unclear [5].

PSC itself is an uncommon but severe disorder; population-based studies report a pooled incidence in the range of 0 to 1.58 per 100,000 person-years, with a mean/median estimate of 0.7–0.8 per 100,000 person-years [6], and a natural history marked by progressive biliary fibrosis, recurrent cholangitis, and eventual cirrhosis or cholangiocarcinoma in a substantial minority of patients [1]. The great majority of PSC occurs alongside IBD, chiefly ulcerative colitis, and this IBD association is itself understood to influence colorectal cancer surveillance needs and long-term hepatic prognosis [1]. Celiac disease, by contrast, is far more prevalent in the general population (estimated at approximately 1% worldwide) but has traditionally been considered a distinct enteropathy with a separate genetic architecture, even though it shares the same HLA-DQ2/DQ8 predisposing haplotypes implicated in other PSC-associated autoimmune conditions [3, 4]. Population-level data quantifying how much more often CeD and PSC co-occur than chance would predict have only recently become available, and, to our knowledge, no prior study has benchmarked the hepatic outcomes of patients with this overlap against outcome data from patients with PSC alone or PSC with IBD but without celiac disease. This absence of a comparator-controlled outcomes analysis has left open the basic clinical question of whether celiac disease overlap meaningfully changes the trajectory of PSC-related liver disease, or whether the apparent severity of reported cases instead reflects small, uncontrolled case series and referral bias.

This retrospective multi-institutional study addresses this gap in two parts. First, we characterize the clinical presentation, disease course, and outcomes of patients with concurrent PSC and CeD, including those with concomitant UC, in a cohort identified across three data sources. Second, to determine whether this overlap is associated with a distinct clinical trajectory rather than simply reflecting the natural history of PSC, we benchmark these outcomes against two independently identified comparator cohorts drawn from the same registry and matched 3:1 to the primary cohort on sex and approximate age at PSC diagnosis: patients with PSC and IBD without celiac disease, and patients with PSC alone without IBD or celiac disease. Together, these analyses aim to address a gap in the literature and to clarify whether increased clinical vigilance, and a specific need for autoimmune cross-screening, is warranted in this rare disease combination.

## Methods

### Study Design and Participant Selection

We conducted a retrospective analysis using electronic health records from three sources: the Mass General Brigham Research Patient Data Registry (RPDR; >7.5 million patients), the Celiac Center at Beth Israel Deaconess Medical Center (BIDMC), and the All of Us Research Program (~ 630,000 participants), a worldwide NIH-managed cohort. Each source provided structured EHR data including demographics, diagnostic codes, laboratory results, and treatment histories.

Eligible patients had confirmed diagnoses of both primary sclerosing cholangitis (PSC) and celiac disease (CeD), defined by ICD-10 codes (encounter threshold > 1, to minimize rule-out diagnoses or coding errors) and validated by clinical, serologic, or histologic evidence in the medical record; RPDR-identified cases additionally underwent manual chart review to confirm diagnostic accuracy. Patients were excluded for secondary sclerosing cholangitis or incomplete diagnostic confirmation of either condition.

### Data Collection and Variables

Extracted data included age at diagnosis, sex, and ethnicity. Age at diagnosis was recorded for both PSC and CeD, and the interval between diagnoses (age at PSC diagnosis minus age at CeD diagnosis) was calculated to characterize temporal sequence. Gluten-free diet adherence was obtained from clinical documentation, given its relevance as a modifiable management factor.

To assess whether PSC-CeD co-occurrence exceeded chance expectations, we calculated the expected number of overlapping cases assuming independence: (N_ce_*d* × N_ps_*c*)/N_total_, where N_ps_*c* and N_ce_*d* are the numbers of patients with PSC and CeD, respectively, and N_total_ is the total registry population. Odds ratios (OR) and 95% confidence intervals (CI) were calculated to quantify and test the significance of this association.

Primary outcome measures included major hepatic complications such as the development of cirrhosis, hepatic decompensation, or cholangiocarcinoma, as well as time to liver transplantation, measured from the date of PSC diagnosis. Secondary outcomes included characterization of triad phenotyping, defined as the simultaneous presence of PSC, CeD, and inflammatory bowel disease (IBD), which represents a distinct clinical entity with potentially unique pathophysiologic mechanisms. Additionally, the diagnostic sequence specifically, the temporal order in which PSC and CeD were diagnosed, was analyzed to determine whether one condition typically preceded the other and whether this sequence influenced clinical outcomes. These outcome metrics were selected to represent clinically meaningful endpoints relevant to patient care and to facilitate a comprehensive assessment of how coexisting celiac disease may influence the natural history, disease progression, and clinical management of PSC.

### Comparator Cohorts and Statistical Analysis

Two comparator cohorts were identified consecutively from the same registry, matched 3:1 on age at PSC diagnosis and gender, without pre-selecting for clinical course: patients with PSC and IBD but without CeD (PSC + IBD, n = 54), and patients with PSC alone (PSC-only, n = 54). Demographic, medication, ALP, and transplant data were extracted from structured EPIC fields and problem-list text; hepatic complications (cirrhosis, fibrosis, autoimmune hepatitis overlap, and portal hypertension-related complications including varices and ascites) were ascertained by manual chart review, consistent with the primary cohort.

Continuous variables (age, ALP) were compared across the three cohorts by one-way ANOVA or Kruskal-Wallis test, with pairwise comparisons by Student’s t-test or Mann-Whitney U test. Categorical variables (sex, race, ethnicity, transplant, biologic use, ursodiol use, hepatic complications) were compared by chi-square test, with pairwise comparisons by Fisher's exact test. Normality of continuous variables was assessed with the Shapiro-Wilk test; ALP values departed significantly from a normal distribution in both matched comparator cohorts (p < 0.0001), so the non-parametric Kruskal-Wallis and Mann-Whitney U tests were used for all ALP comparisons. Given the exploratory nature of these pairwise comparisons across multiple outcomes and cohorts, p-values were not adjusted for multiple comparisons; results of individual pairwise tests should therefore be interpreted as hypothesis-generating rather than confirmatory. Treatment-stratified subgroup counts (biologic and ursodiol exposure vs. transplant and cirrhosis, within each cohort) are reported descriptively only, without formal significance testing, given the small subgroup sizes involved. Two-tailed p < 0.05 was considered significant.

### Ethics Statement

This study was approved by the Institutional Review Board of Mass General Brigham, Boston, Massachusetts, USA. All research was conducted in accordance with the ethical principles outlined in the Declaration of Helsinki and Istanbul. As a retrospective study, informed consent was waived by the IRB under this protocol. (Protocol #:2024P002811)

### Declaration of the use of AI

During the preparation of this manuscript, the authors used GPT-5.3 to assist with grammar checking, spelling, and sentence optimization to improve readability. This tool was not used to generate scientific insights, analyze or interpret data, or draw conclusions. All AI-assisted content was reviewed, edited, and approved by the authors, who take full responsibility for the accuracy and integrity of the published work.

### Data Availability Statement

The data that support the findings of this study are available from the Research Patient Data Registry (RPDR) and the All of Us Research Program but restrictions apply to the availability of these data, which were used under license for the current study, and so are not publicly available. De-identified data are however available from the authors upon reasonable request and with permission of the respective institutions' data access committees.

## Results

Among 7.5 million RPDR patients, 18,375 had celiac disease and 992 had PSC. Expected overlap by chance was 2.43 cases; 13 were observed in RPDR (OR 5.35, 95% CI 3.08–9.30; p < 0.001), indicating significantly increased co-occurrence. An additional 5 cases were identified from other data sources, bringing the total to 18 patients with dual PSC and celiac disease.

Baseline demographic characteristics and IBD distribution are summarized in (Table 1). The cohort included 12 males and 6 females, with a mean age of 47.8 years at PSC diagnosis and 44.4 years at celiac disease diagnosis. Most patients were White (94%) and non-Hispanic (89%). Concomitant inflammatory bowel disease (IBD) was present in 13 patients (72.2%), most commonly ulcerative colitis (10 patients, 55.6%), followed by Crohn’s disease (2 patients, 11.1%) and indeterminate colitis (1 patient, 5.6%). Among the two patients with Crohn’s disease, one had ileocolonic involvement and one had upper gastrointestinal involvement with ileal disease. Five patients (27.8%) had no documented IBD. Gluten-free diet adherence was documented in 16 patients (88.9%).

### Medication exposure

Medication exposure is summarized in (Table 2a). Ursodiol was the most commonly prescribed agent (55.6%), often continued for over a decade, consistent with standard PSC management. Mesalamine was used by 55.6% for UC control, frequently for ≥ 15 years. Tacrolimus (22.2%) and azathioprine (27.8%), as well as biologic agents including adalimumab, infliximab, vedolizumab, ustekinumab, golimumab, and risankizumab, were used across the cohort. Escalation to biologic therapy (n = 7) occurred in patients with moderate to severe disease or treatment-refractory IBD.

### Clinical course

Hepatic outcomes, transplantation, and follow-up are summarized in (Table 3). Mean follow-up from PSC diagnosis was 11.53 years. Hepatic complications occurred in 16 patients (88.9%), with cirrhosis (including portal hypertension, autoimmune hepatitis overlap, and post-transplant cases) developing in 9 patients (50.0%), including 4 patients (22.2%) with PSC–autoimmune hepatitis overlap. Fibrosis without cirrhosis was observed in 3 patients (16.7%). Two patients (11.1%) had no documented hepatic complications, and data were incomplete for 2 patients (11.1%).

Transplantation and follow-up characteristics are summarized in (Table 4). Liver transplantation was performed in 4 patients (22.2%), with a mean interval from PSC diagnosis to transplant of 5.39 years (n = 3 with dated intervals, range 3.1–8.1 years; a date was unavailable for the remaining transplant). No recurrence of PSC was observed following transplantation during the available follow-up period.

### Disease Duration and Progression in Non-Transplanted Patients

Among the 14 non-transplanted patients, the mean PSC disease duration was approximately 8.8 years, with a range between 2 and 24 years. Approximately 79% of these non-transplanted patients adhered to a gluten-free diet. Cirrhosis was present in about 29% of this group, either before or during follow-up.

### Temporal Sequence of Diagnosis

The sequence of disease onset varied considerably (Fig. 1). Among the PSC + CeD cohort (n = 18), IBD was diagnosed first in 7 patients (38.9%), celiac disease (CeD) first in 7 patients (38.9%), and PSC first in 3 patients (16.7%); the order could not be determined in the remaining patient (5.6%).

Among the 7 patients with IBD first: IBD → CeD occurred in 4 (mean interval 4.8 years), of whom 3 went on to PSC (mean interval 2.9 years); IBD → PSC occurred in 3 (mean interval 4.4 years), all of whom went on to CeD (mean interval 4.7 years).

Among the 7 patients with CeD first: CeD → PSC occurred in 5 (mean interval 3.4 years), of whom 2 went on to IBD (mean interval 12.5 years); CeD → IBD occurred in 2 (mean interval 4.3 years), both of whom went on to PSC (mean interval 7.7 years).

Among the 3 patients with PSC first: PSC → CeD occurred in all 3 (mean interval 2.2 years), with 1 going on to IBD (interval 11.0 years).

### Disease Severity

Patients with coexisting PSC, celiac disease (CeD), and ulcerative colitis (UC) were followed longitudinally for variable durations. All patients with CeD had documented adherence to a gluten-free diet during follow-up.

Hepatobiliary complications, including hepatic fibrosis, cirrhosis, portal hypertension, recurrent cholangitis, and liver transplantation, were observed across the cohort (Table 3). Cirrhosis and advanced liver disease occurred in patients with both well-controlled and active intestinal disease states.

UC management included mesalamine maintenance therapy in most patients, with escalation to immunosuppressive or biologic agents in those with more extensive or refractory disease (Table 2a). PSC-related complications were observed across UC disease activity and treatment categories.

### Alkaline Phosphatase Trends

Longitudinal alkaline phosphatase (ALP) trends are summarized in (Tables 5a, 5b). Relative to celiac disease diagnosis, 16.7% of patients (n = 3) had a mean ALP value recorded before diagnosis (mean 178.8 U/L) and 66.7% (n = 12) had a mean ALP value recorded after diagnosis (mean 198.3 U/L). Relative to PSC diagnosis, 16.7% of patients (n = 3) had a mean ALP value recorded before diagnosis (mean 155.4 U/L) and 61.1% (n = 11) had a mean ALP value recorded after diagnosis (mean 317.4 U/L).

Among patients with paired pre- and post-diagnosis values, mean ALP rose from 162.9 U/L to 173.3 U/L overall; 50% of patients showed an increase and 50% showed a decrease. Individual ALP values ranged from 53.3 to 1119.8 U/L across all measurements, with the highest values concentrated in the post-PSC-diagnosis period, consistent with progressive cholestatic injury following PSC onset in this cohort.

### Comparison with Matched PSC Control Cohorts

To contextualize these findings, the PSC + CeD cohort (± IBD; n = 18) was compared with two control cohorts drawn from the same registry and approximately matched on age at PSC diagnosis: patients with PSC and IBD but without celiac disease (PSC + IBD, n = 54) and patients with PSC alone (PSC-only, n = 54) (Table 1). Age at PSC diagnosis, sex, and ethnicity were broadly similar across the three cohorts. Mean follow-up from PSC diagnosis was 11.59 years (range 2.0–27.6) in the PSC + IBD controls and 8.46 years (range 0.02–18.44) in the PSC-only controls.

Liver transplantation was numerically more frequent in the PSC + CeD cohort (22.2%) than in PSC + IBD controls (5.6%) or PSC-only controls (13.0%), though this difference did not reach significance (chi-square p = 0.127) (Table 4, Fig. 3). Cirrhosis was likewise more common in the PSC + CeD cohort and in PSC-only controls than in PSC + IBD controls (50.0% and 51.9% vs 16.7%; chi-square p = 0.0002), fibrosis without cirrhosis was more common in the PSC + CeD and PSC + IBD cohorts than in PSC-only controls (16.7% and 18.5% vs 0%; p = 0.005), and PSC–autoimmune hepatitis overlap was most frequent in the PSC + CeD cohort, intermediate in PSC + IBD controls, and absent in PSC-only controls (22.2% vs 9.3% vs 0%; chi-square p = 0.005) (Table 3, Fig. 4). Any hepatic complication was numerically most common in the PSC + CeD cohort (88.9% vs 63.0% PSC + IBD and 61.1% PSC-only), though this difference did not reach significance (p = 0.083). Hepatic complications and cirrhosis in the two control cohorts were ascertained by manual chart review, consistent with the approach used for the PSC + CeD cohort (Tables 1–5b).

ALP levels differed significantly across the three cohorts at both the pre-diagnosis and post-diagnosis/1-year time points (Kruskal-Wallis p = 0.0019 and p = 0.0087, respectively). Pre-diagnosis ALP was highest in PSC-only controls (mean 245.6 U/L, n = 43), followed by the PSC + CeD cohort (155.4 U/L, n = 3) and PSC + IBD controls (138.7 U/L, n = 24). Post-diagnosis ALP, available for 11 of 18 PSC + CeD patients (mean 317.4 U/L), was numerically higher than both PSC-only controls (262.3 U/L, n = 33) and PSC + IBD controls (166.3 U/L, n = 35). Case-cohort ALP data remain more limited than the control cohorts and should be interpreted with caution (Table 5a, Fig. 2).

Biologic use was numerically similar across the three cohorts (38.9% PSC + CeD vs 37.0% PSC + IBD controls vs 22.2% PSC-only controls) and did not differ significantly (chi-square p = 0.183). Ursodiol use, by contrast, was significantly lower in the PSC + CeD cohort (55.6%) than in PSC + IBD controls (70.4%) and PSC-only controls (85.2%) (chi-square p = 0.028) (Table 2b).

Time from PSC diagnosis to cirrhosis diagnosis trended later in the PSC + CeD cohort (mean 3.67 years, n = 3) than in either control cohort, where cirrhosis was typically detected at or near the time of PSC diagnosis (PSC + IBD mean 0.87 years, n = 10; PSC-only mean − 0.08 years, n = 25; Kruskal-Wallis p = 0.058) (Table 6, Fig. 5). Given the small case-cohort sample, this later apparent timing should be regarded as a preliminary signal rather than a confirmed finding.

All 18 PSC + CeD patients had documented gluten-free diet (GFD) status; 14 (77.8%) were recorded as adherent and the remaining 4 (22.2%) as “not known,” with no patient documented as non-adherent. Because no true non-adherent group was available for comparison, GFD adherence could not be formally tested against outcomes; descriptively, transplant occurred in 3/14 (21.4%) adherent versus 1/4 (25.0%) unknown-adherence patients, and cirrhosis in 6/14 (42.9%) versus 2/4 (50.0%), respectively (both Fisher’s exact p = 1.00).

Within each cohort, outcomes were also examined descriptively by treatment exposure (biologic use and ursodiol use) against liver transplant and cirrhosis (Table 7). Transplant and cirrhosis counts were broadly similar between exposed and unexposed patients within each cohort, with no consistent pattern favoring either exposure group. Given the small subgroup sizes throughout (e.g., only 2 biologic-exposed PSC-only patients), these counts are reported descriptively rather than tested statistically and should not be read as evidence for or against a treatment effect.

## Discussion

This study provides one of the largest multi-institutional characterizations to date of patients with coexisting PSC, celiac disease, and ulcerative colitis. We found a significant enrichment of co-occurrence among these three immune-mediated disorders, well beyond what would be expected by chance, supporting a shared susceptibility rather than a coincidental clustering. Prior literature on this overlap has been limited to case reports and small series [2][4]; this analysis extends that work with outcome data from a more systematically identified cohort. That said, given the rarity of this combination and the retrospective, registry-based ascertainment, the magnitude of enrichment should be interpreted cautiously, as referral and detection bias likely inflate the apparent association to some degree.

Diagnostically, IBD and celiac disease were about equally likely to be the first condition identified, while PSC itself was rarely the presenting diagnosis, consistent with gastrointestinal or malabsorptive symptoms typically prompting workup before any hepatobiliary involvement is recognized. Notably, the interval between IBD and PSC diagnosis was far shorter in this cohort than in matched PSC + IBD controls, where IBD typically preceded PSC by over a decade, suggesting that celiac disease overlap may be associated with a more compressed, near-simultaneous multi-diagnosis presentation rather than the usual sequential pattern. Taken together with the known shared HLA-DR3/DQ2 susceptibility across these conditions, these findings support a pattern of immune-mediated clustering along the gut-liver axis [4][5].

With near-universal adherence to a gluten-free diet, liver-related morbidity remained substantial. Most patients developed at least one major hepatic complication, and roughly one in five required liver transplantation, at a burden at least as high as that reported in classical PSC cohorts [1]. This did not, however, translate into a clearly accelerated path to cirrhosis; if anything, cirrhosis tended to be identified later relative to PSC diagnosis than in either comparator cohort. Together, these observations suggest celiac disease overlap may be associated with a greater cumulative burden of hepatic complications and transplantation rather than a uniformly faster disease course, though this should be read as a preliminary signal given the small sample, potential referral bias, and the very limited number of patients with dateable cirrhosis onset in this cohort.

Benchmarking against the matched comparator cohorts helps localize where this excess burden concentrates. Liver transplantation and cirrhosis were both more common in the PSC + CeD cohort than in PSC + IBD controls, but cirrhosis rates in the PSC + CeD cohort closely resembled those in PSC-only controls, who also lack IBD. This raises the possibility that the presence of IBD itself may track with a comparatively milder hepatic course in PSC, and that the elevated hepatic burden seen with celiac overlap could partly reflect the absence of IBD rather than a specific effect of celiac disease, a distinction that will require larger, prospectively matched cohorts to resolve. The markedly higher rate of PSC-autoimmune hepatitis overlap in the PSC + CeD cohort, by contrast, does point toward a more specific link between celiac disease and overlapping autoimmune liver injury, consistent with the shared HLA-DR3/DQ2 susceptibility noted above.

In contrast to prior reports suggesting gluten-free diet improves gastrointestinal symptoms without meaningfully altering hepatic outcomes [9], our study offers a more comprehensive view of disease trajectory, incorporating diagnostic sequence and treatment exposure. Given the small number of transplanted patients with celiac disease in this cohort, no firm conclusions can be drawn about post-transplant course. Anecdotally, none developed recurrent PSC during follow-up, though this observation is far too limited to support any inference about whether GFD adherence modifies post-transplant recurrence risk, a phenomenon reported in a meaningful minority of transplanted PSC patients without celiac disease, and one that remains a worthwhile question for future prospective study [7][8].

The sequence of diagnoses was notably heterogeneous, with no single trajectory clearly dominant across the cohort; patients arrived at this triad by varied and sometimes closely spaced pathways rather than a single classical progression. This underscores the need for vigilant cross-screening: persistent gastrointestinal or nutritional symptoms in PSC or IBD should prompt evaluation for celiac disease [2][5], while abnormal liver biochemistry or bowel symptoms in celiac disease warrant hepatobiliary and colonic assessment [1].

Mechanistically, our findings support the hypothesis that shared immune pathways, potentially mediated through common HLA-associated antigen presentation, underlie this disease constellation [4][5]. Clinical trajectories, however, still appeared largely driven by the dominant condition: PSC shaping hepatic outcomes [1], celiac disease shaping malabsorption [3], and UC shaping colonic inflammation, with limited evidence that treating one condition consistently altered the course of the others. This reinforces the need for individualized, multidisciplinary management rather than assuming a single unifying disease course.

Clinically, these findings suggest a need for heightened awareness of occult celiac disease in PSC patients, particularly those with UC, and reciprocal awareness of PSC in celiac patients with abnormal liver tests [2][5]. While gluten-free diet adherence was associated with improvement in celiac-related symptoms, it did not appear to alter PSC progression in most cases [9]. The absence of post-transplant recurrence among transplanted patients in this cohort is worth noting but, given the small numbers and limited follow-up, should be interpreted cautiously and requires validation in larger studies [7][8].

The strengths of this study include its multi-institutional design, rigorous diagnostic confirmation, and extended follow-up; incorporating matched PSC + IBD and PSC-only control cohorts also addressed the lack of a comparator group inherent to the primary cohort alone. Limitations are nonetheless substantial: treatment-stratified subgroups were too small to support formal statistical comparison and are reported descriptively only, gluten-free diet adherence could not be tested against outcomes because no clearly non-adherent patients were identified for comparison, and the retrospective, modest-sized primary cohort remains susceptible to referral and documentation bias. Genotypic and environmental exposure data were also incomplete. These limitations temper generalizability, and larger, prospective, population-based studies incorporating immunophenotyping and environmental exposures are needed to confirm and extend these findings.

In conclusion, this study provides further evidence for a clinically significant overlap between PSC, celiac disease, and UC. The high burden of hepatic complications observed, alongside the absence of post-transplant recurrence in GFD-adherent patients, underscores both the clinical significance of this association and the need for further mechanistic and therapeutic investigation [1][5]. These findings support integrated, multidisciplinary care and provide a framework for future work exploring gut-liver immune interactions in autoimmune disease.

## Figures and Tables

**Figure 1:**
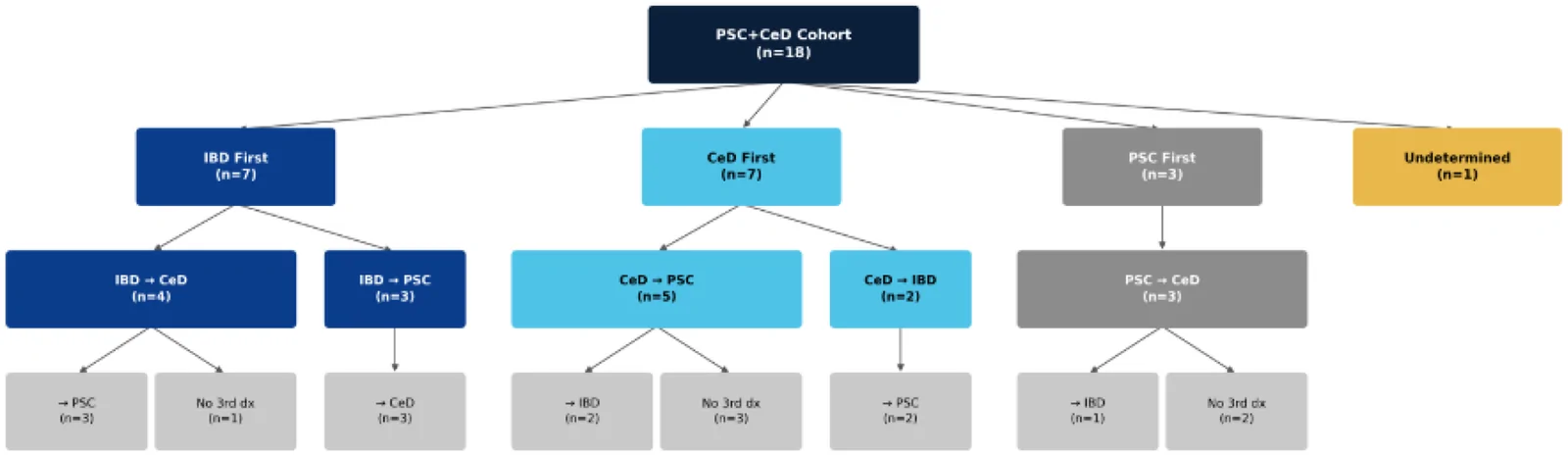
Temporal sequence of PSC, celiac disease, and IBD diagnosis in the PSC+CeD cohort. Hierarchical diagram tracing each patient’s (n = 18) diagnostic order across primary sclerosing cholangitis (PSC), celiac disease (CeD), and inflammatory bowel disease (IBD). The top tier shows which condition was diagnosed first; subsequent tiers show the second and third/final diagnoses reached by patients in each branch, with case counts (n) labeled in every box. One patient’s diagnostic sequence could not be reconstructed and is shown separately (“Undetermined”).

**Figure 2:**
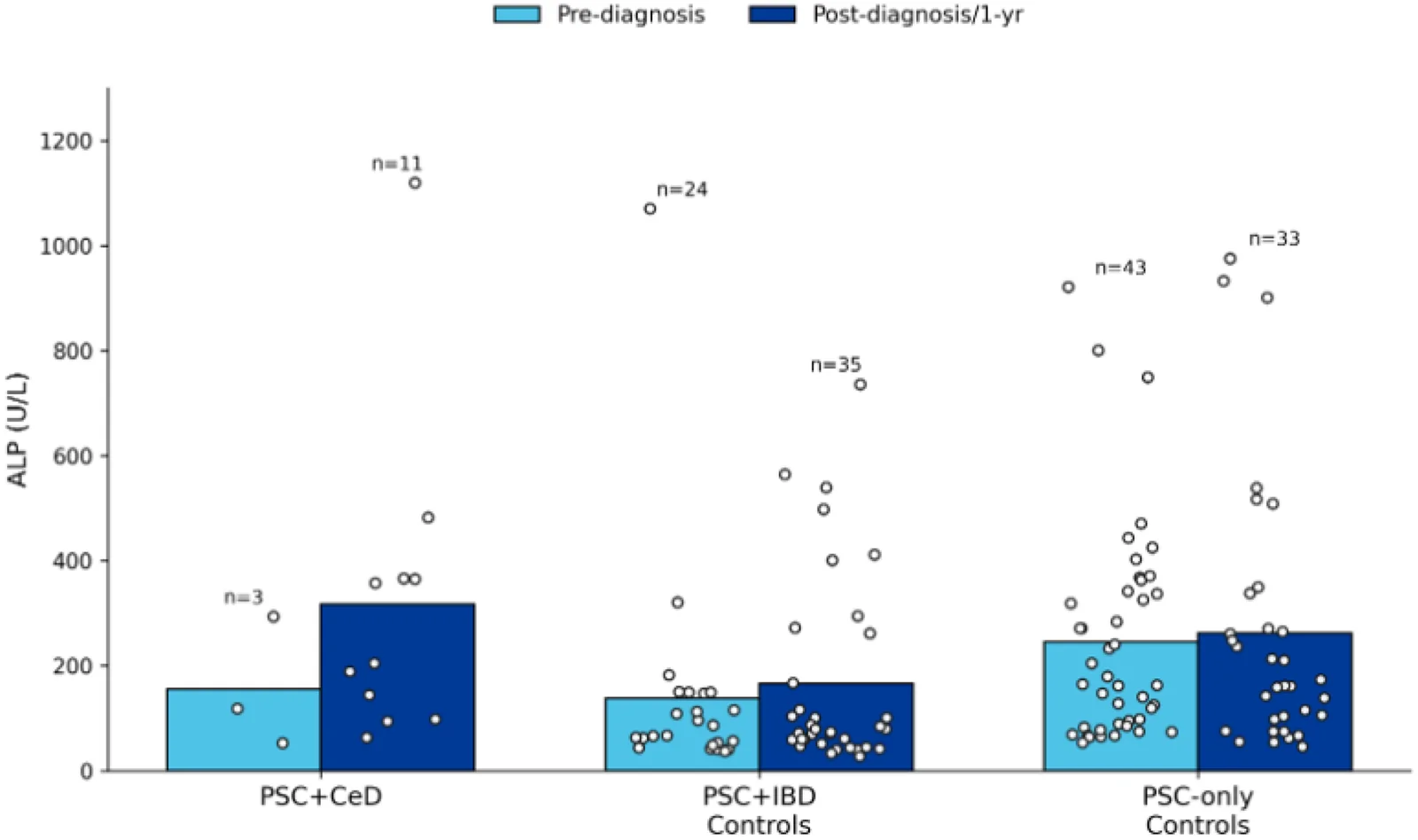
Alkaline phosphatase (ALP) levels before and after PSC diagnosis, by cohort. Bars show mean ALP (U/L) at the pre-diagnosis and post-diagnosis/1-year time points for the PSC+CeD cohort (pre-diagnosis n = 3, post-diagnosis n = 11) and the two matched comparator cohorts, PSC+IBD controls (n = 24, n = 35) and PSC-only controls (n = 43, n = 33); open circles show individual patient-level values, jittered horizontally for visibility. ALP differed significantly across the three cohorts at both time points (Kruskal-Wallis p = 0.0019 pre-diagnosis; p = 0.0087 post-diagnosis/1-year). The small pre-diagnosis case-cohort sample limits precision and should be interpreted with caution.

**Figure 3:**
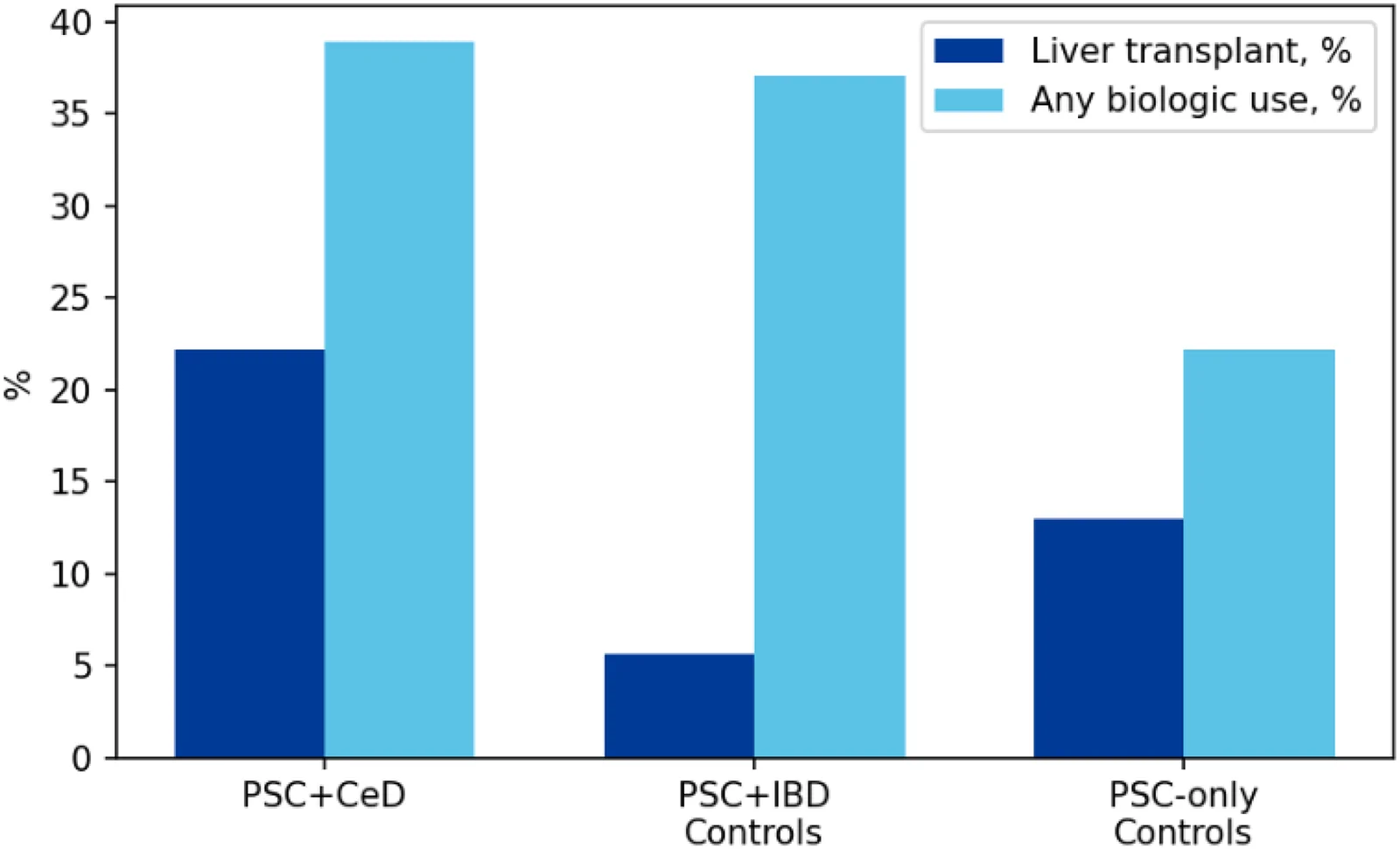
Liver transplantation rate and biologic use, by cohort. Bars show the proportion of patients (%) who underwent liver transplantation and the proportion with any biologic exposure, for the PSC+CeD cohort (n = 18) and the two matched comparator cohorts, PSC+IBD controls (n = 54) and PSC-only controls (n = 54). Neither difference reached statistical significance (transplantation, chi-square p = 0.127; biologic use, chi-square p = 0.183).

**Figure 4:**
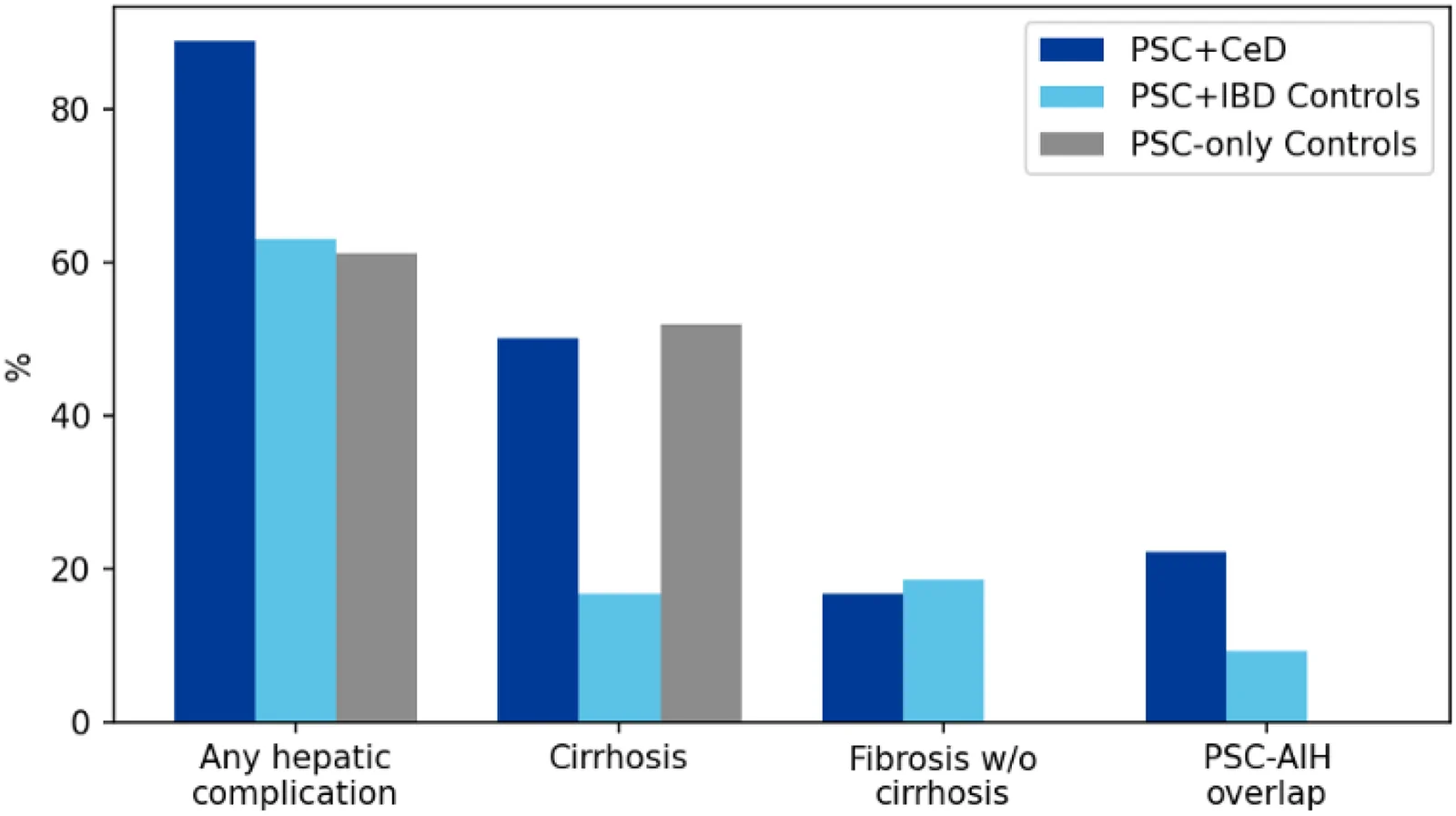
Hepatic complications, by cohort. Bars show the proportion of patients (%) with any hepatic complication, cirrhosis, fibrosis without cirrhosis, and PSC–autoimmune hepatitis (AIH) overlap, for the PSC+CeD cohort (n = 18) and the two matched comparator cohorts, PSC+IBD controls (n = 54) and PSC-only controls (n = 54). Cirrhosis (chi-square p = 0.0002), fibrosis without cirrhosis (p = 0.005), and PSC-AIH overlap (p = 0.005) differed significantly across cohorts; the overall rate of any hepatic complication did not (p = 0.083).

**Figure 5:**
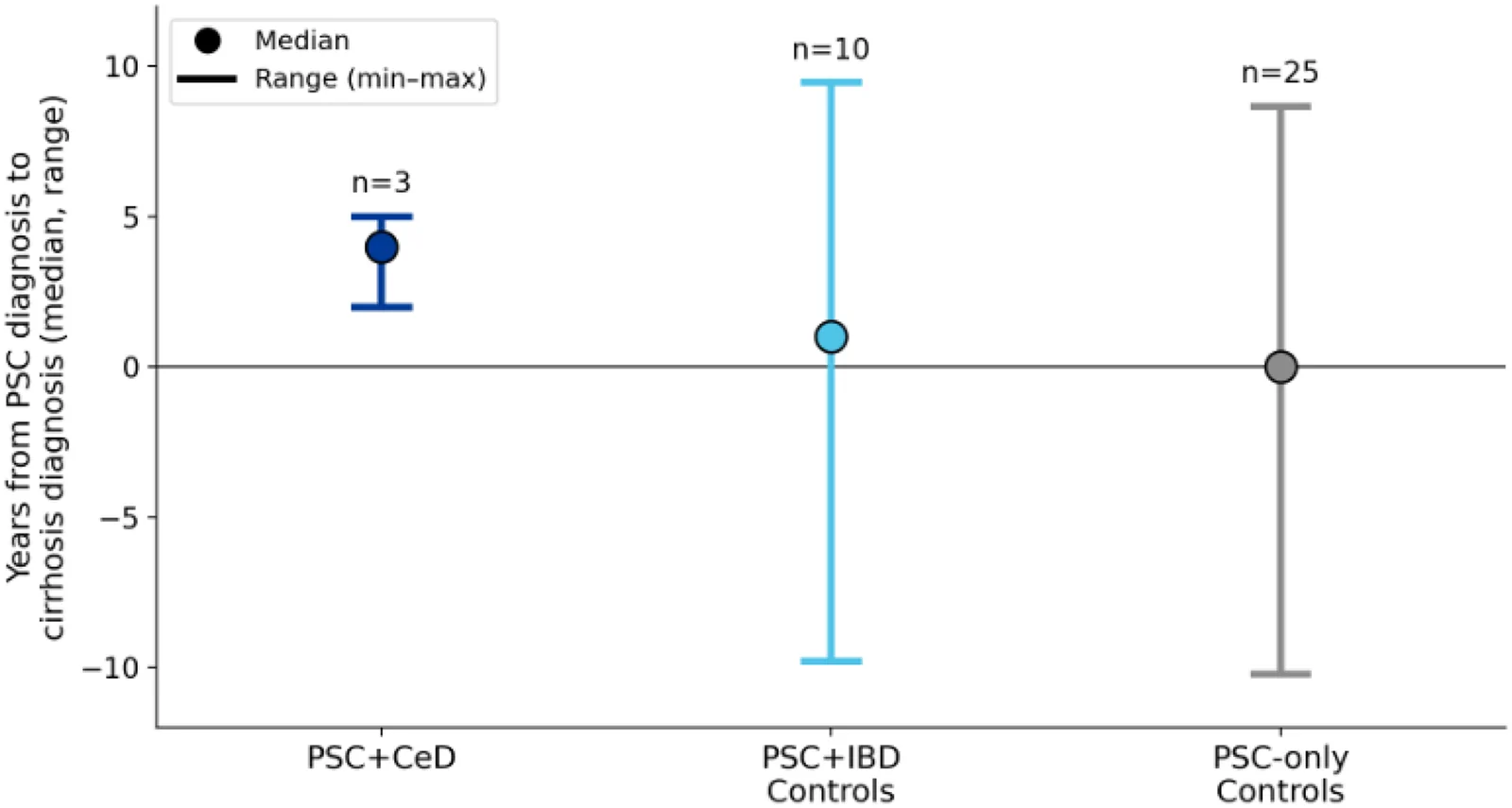
Time from PSC diagnosis to cirrhosis diagnosis, by cohort. Points show median years from PSC diagnosis to cirrhosis diagnosis, with vertical lines indicating the full range (minimum–maximum), for patients with a documented cirrhosis diagnosis, for the PSC+CeD cohort (n = 3), PSC+IBD controls (n = 10), and PSC-only controls (n = 25). Negative values indicate cirrhosis diagnosed before the recorded PSC diagnosis date. Cirrhosis was diagnosed later relative to PSC diagnosis in the PSC+CeD cohort than in either comparator cohort (Kruskal-Wallis p = 0.058); given the small case-cohort sample, this should be regarded as a preliminary signal.

**Table 1:** Demographics and baseline characteristics, by cohort

Characteristic	PSC + CeD (n = 18)	PSC + IBD Controls (n = 54)	PSC-only Controls (n = 54)	p-value
Age at PSC diagnosis, mean (yrs)	47.8	39.0	34.2	0.161 (ANOVA)
Age at CeD diagnosis, mean (yrs)	44.4	n/a	n/a	—
Sex, M:F	12:6	37:17	36:18	—
Race, White, n (%)	17 (94.4)	42 (77.8)	45 (83.3)	—
Ethnicity, Non-Hispanic, n (%)	16 (88.9)	44 (81.5)	43 (79.6)	—
Ethnicity, Hispanic, n (%)	1 (5.6)	2 (3.7)	2 (3.7)	—
Ethnicity, Unknown/Declined, n (%)	1 (5.6)	8 (14.8)	9 (16.7)	—
IBD present, n (%)^a^	13 (72.2)	54 (100)	n/a	—
Ulcerative colitis	10 (55.6)	37 (68.5)	n/a	—
Crohn's disease	2 (11.1)	17 (31.5)	n/a	—
Indeterminate colitis	1 (5.6)	1 (1.9)	n/a	—
IBD absent/unknown	5 (27.8)	0 (0)	n/a	—
Mean follow-up from PSC diagnosis, yrs (range)	11.53	11.59 (2.0–27.6)	8.46 (0.02–18.44)	0.132 (K-W)

a IBD is present by definition in 100% of PSC + IBD controls (matching criterion); not applicable to PSC-only controls.

**Table 2 a.** Medication exposure in the PSC + CeD cohort (n = 18)

Category	Medication	n	%	Longest duration of use
Conventional agents	Ursodiol	10	55.6	≥ 15 years
Conventional agents	Mesalamine	10	55.6	≥ 16 years
Immunosuppressants	Tacrolimus	4	22.2	Up to 8 years or ongoing
Immunosuppressants	Azathioprine	5	27.8	Up to 8 years or ongoing
Biologics (combined)	Adalimumab, infliximab, vedolizumab, ustekinumab, golimumab, risankizumab^a^	7	38.9	Up to 7 years (some recent starts 20232025)

a Any of the listed agents; patients counted once regardless of the number of biologic agents used.

**Table 2 b.** Biologic and ursodiol use, by cohort

Medication class	PSC + CeD (n = 18)	PSC + IBD Controls (n = 54)	PSC-only Controls (n = 54)	p-value
Any biologic use, n (%)	7 (38.9)	20 (37.0)	12 (22.2)	0.183 (chi-sq)
Ursodiol use, n (%)	10 (55.6)	38 (70.4)	46 (85.2)	0.028 (chi-sq)

**Table 3:** Hepatic complications, by cohort

Complication	PSC + CeD (n = 18)	PSC + IBD Controls (n = 54)	PSC-only Controls (n = 54)	p-value
Any hepatic complication, n (%)	16 (88.9)	34 (63.0)	33 (61.1)	0.083 (chi-sq)
Cirrhosis (± portal HTN, ± AIH overlap, incl. post-transplant), n (%)^a^	9 (50.0)	9 (16.7)	28 (51.9)	0.0002 (chi-sq)
Fibrosis without cirrhosis, n (%)	3 (16.7)	10 (18.5)	0 (0)	0.005 (chi-sq)
PSC-AIH overlap, n (%)^a^	4 (22.2)	5 (9.3)	0 (0)	0.005 (chi-sq)
No hepatic complications documented, n (%)^b^	2 (11.1)	n/a	n/a	—
Data incomplete, n (%)^b^	2 (11.1)	n/a	n/a	—

a Cirrhosis, fibrosis without cirrhosis, and PSC–AIH overlap are not mutually exclusive categories. In the PSC + CeD cohort, the three counts sum to the “any hepatic complication” total (16); in the comparator cohorts they do not, indicating additional complication types (e.g., recurrent cholangitis, decompensation, cholangiocarcinoma) not separately itemized here.

b Case cohort only; not separately ascertained in the matched comparator cohorts.

**Table 4:** Liver transplantation, by cohort

Metric	PSC + CeD (n = 18)	PSC + IBD Controls (n = 54)	PSC-only Controls (n = 54)	p-value
Liver transplantation performed, n (%)	4 (22.2)	3 (5.6)	7 (13.0)	0.127 (chi-sq)
Mean interval, PSC diagnosis → transplant, yrs	5.39 (n = 3, range 3.1–8.1)	3.91 (n = 2, range 3.5–4.3)	5.31 (n = 6, range − 0.2–14.0)	—

**Table 5 a.** Alkaline phosphatase (ALP) trends anchored to PSC diagnosis, by cohort

Metric	PSC + CeD (n = 18)	PSC + IBD Controls (n = 54)	PSC-only Controls (n = 54)	p-value
Pre-diagnosis, mean U/L (n)	155.4 (n = 3)	138.7 (n = 24)	245.6 (n = 43)	0.0019 (K-W)
Post-diagnosis/1-yr, mean U/L (n)	317.4 (n = 11)	166.3 (n = 35)	262.3 (n = 33)	0.0087 (K-W)

**Table 5 b.** ALP anchored to celiac disease diagnosis, PSC + CeDcohort only (n = 18)

Metric	n/18 (%)	Mean ALP (U/L)
Before celiac diagnosis	3 (16.7)	178.8
After celiac diagnosis	12 (66.7)	198.3

**Table 6:** Time from PSC diagnosis to cirrhosis diagnosis

Cohort	n	Mean (yrs)	Median (yrs)	Range (yrs)
PSC + IBD Controls	10	0.87	1.01	−9.78 to 9.46
PSC-only Controls	25	−0.08	0.02	−10.22 to 8.66
PSC + CeD	3	3.67	4	2.0 to 5.0

**Table 7:** Outcomes stratified by treatment exposure within comparator cohorts

Cohort	Stratifier	Transplant, Yes vs No	Cirrhosis, Yes vs No
PSC + IBD	Any biologic (30 vs 24)	1/30 vs 0/24	6/30 vs 5/24
PSC + IBD	Ursodiol (30 vs 24)	1/30 vs 0/24	7/30 vs 4/24
PSC-only	Any biologic (2 vs 52)	0/2 vs 3/52	1/2 vs 24/52
PSC-only	Ursodiol (37 vs 17)	3/37 vs 0/17	19/37 vs 6/17
PSC + CeD	Any biologic (7 vs 11)	2/7 vs 2/11	4/7 vs 4/11
PSC + CeD	Ursodiol (10 vs 8)	2/10 vs 2/8	4/10 vs 4/8

## Data Availability

The data that support the findings of this study are available from the Research Patient Data Registry (RPDR) and the All of Us Research Program but restrictions apply to the availability of these data, which were used under license for the current study, and so are not publicly available. De-identified data are however available from the authors upon reasonable request and with permission of the respective institutions' data access committees.
